# Temporal fossa arachnoid cyst presenting with bilateral subdural hematoma following trauma: two case reports

**DOI:** 10.1186/1752-1947-3-53

**Published:** 2009-02-09

**Authors:** Promod Pillai, Sajesh K Menon, Raju P Manjooran, Rajiv Kariyattil, Ashok B Pillai, Dilip Panikar

**Affiliations:** 1 Department of Neurological Surgery, the Ohio State University Medical Center, Hamilton Hall, Neil Avenue, Columbus, Ohio 43210, USA; 2 Department of Neurosurgery, Amrita Institute of Medical Sciences, Kochi, India

## Abstract

**Introduction:**

Intracranial arachnoid cysts are considered to be congenital malformations with a predilection for the temporal fossa. They are often asymptomatic but can sometimes be symptomatic due to enlargement or hemorrhage. There are multiple case reports of arachnoid cysts becoming symptomatic with hemorrhagic complications following head trauma. In such cases, the bleeding is often confined to the side ipsilateral to the arachnoid cyst. Occurrence of contralateral subdural hematomas in patients with temporal fossa arachnoid cysts has rarely been observed and is reported less frequently in the medical literature.

**Case presentation:**

We report two cases of people (a 23-year-old man and a 41-year-old man) with temporal fossa arachnoid cysts complicated by a subdural hematoma following head injury. Both patients developed a subdural hematoma contralateral to the side of a temporal fossa arachnoid cyst. It is likely that lack of adequate intracranial cushioning in the presence of an intracranial arachnoid cyst may result in injury not only to ipsilateral but also to contralateral bridging veins, following head trauma.

**Conclusion:**

It is important to identify and report such rare complications with intracranial arachnoid cysts, so that asymptomatic patients with an intracranial arachnoid cyst can be counseled about such possibilities following head trauma.

## Introduction

Arachnoid cysts are believed to be developmental anomalies and are often documented as incidental findings on imaging [1]. A common location is at the temporal fossa [2,3]. Occasionally, these cysts become symptomatic with hemorrhagic complications, often precipitated by head trauma. Hemorrhagic complications are often confined to the side ipsilateral to the location of the arachnoid cyst, and the occurrence of contralateral hemorrhage has been documented on occasion in the literature. We report two patients with temporal fossa arachnoid cysts who experienced contralateral subdural hematomas following head trauma.

## Case presentation

### Case 1

**Figure 1:**
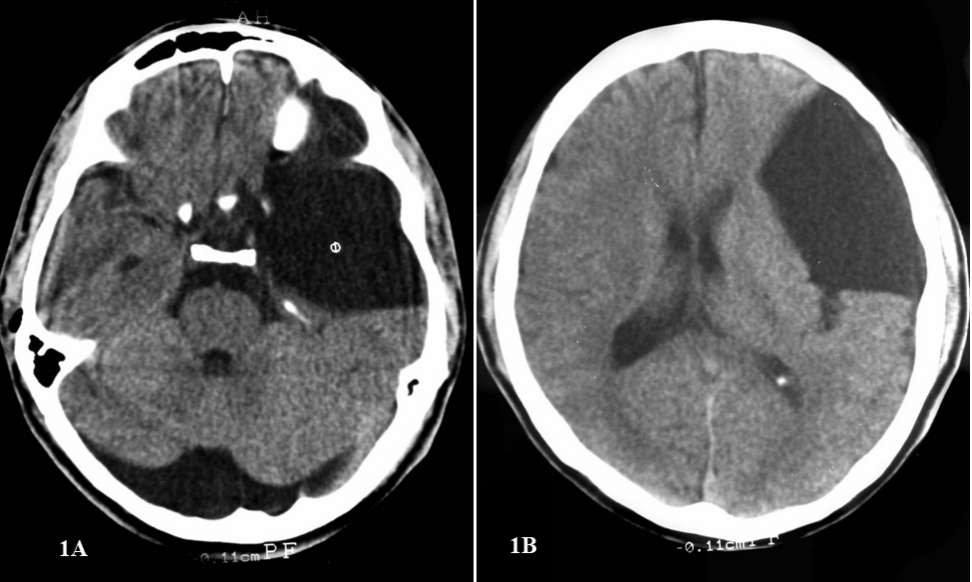
**Computed tomographic scan of the head without contrast immediately following the trauma, showing a giant arachnoid cyst in the middle cranial fossa**.

**Figure 2:**
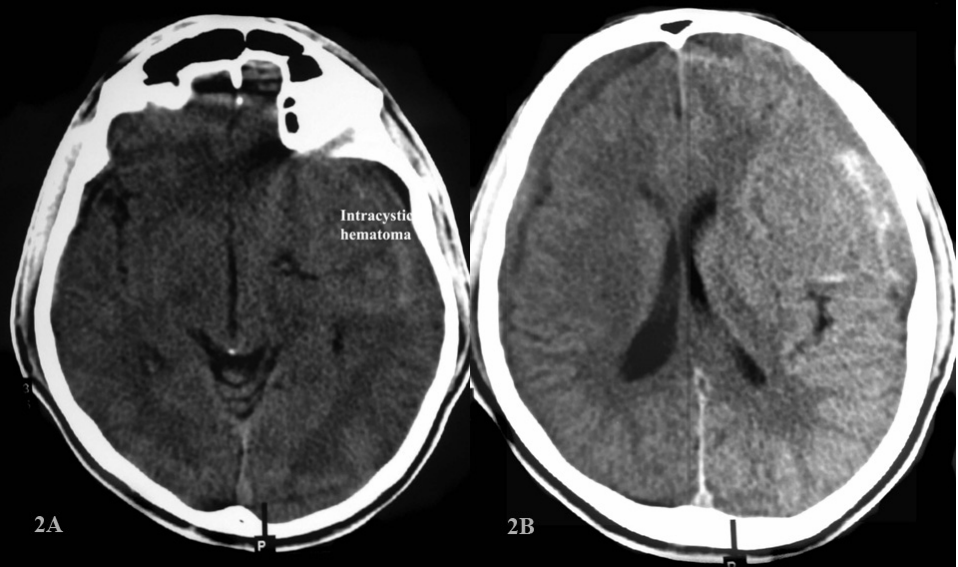
**Computed tomographic scan of the head without contrast 3 weeks after the trauma**. Figure 2A shows an intracystic hematoma. Figure 2B shows a bilateral subdural hematoma of mixed density.

A previously asymptomatic 23-year-old man was examined in the emergency services unit of the referring hospital following a fall while riding a bicycle. He reported no loss of consciousness and no external injuries but was complaining of mild headache and nausea. On examination, the patient was fully conscious and demonstrated no focal neurological deficit. On admission, a computer tomography (CT) scan of his head revealed a giant arachnoid cyst, Galassi type II [4], occupying the left middle cranial fossa and extending into the sylvian fissure (Figure 1). There was no brain parenchymal injury or intracranial hemorrhage. Incidentally, the patient also presented with a mega cisterna magna. He was subsequently discharged. Four weeks later, he presented to our institution with increasing headaches, nausea and vomiting. On admission, the patient was conscious and without any focal neurological deficits. A repeat CT scan of his head revealed a bilateral mixed density subdural hematoma with a mixed density mass lesion within the left middle cranial fossa extending into the sylvian fissure (Figure 2), replacing the previously documented cerebrospinal fluid (CSF) density lesion. The patient underwent a left-sided craniotomy for evacuation of the subdural hematoma as well as the intracystic hematoma and cyst fenestration into the basal cisterns. Right frontal and parietal burr holes were also performed to evacuate the right-sided subdural hematoma. The patient tolerated the procedure well and recovered completely.

### Case 2

**Figure 3:**
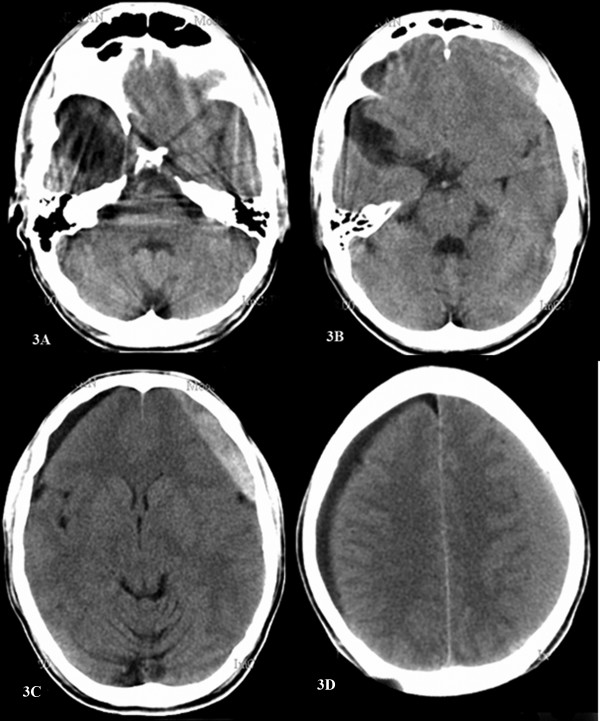
**Computed tomographic scan of the head without contrast showing an arachnoid cyst in the right temporal fossa**. A, B) an ipsilateral subdural hygroma and C, D) a contralateral subdural hematoma.

A 41-year-old man presented to our out-patient unit with an increasingly severe headache and nausea of 2 weeks' duration. He recalled sustaining a trivial fall about 12 weeks prior to presentation, with no loss of consciousness or any external injuries. On admission, the patient was conscious, with no focal neurological deficits. A cranial CT scan revealed a Galassi Type I arachnoid cyst occupying the right temporal fossa (Figure 3A,B) with an ipsilateral subdural hygroma and a contralateral subdural hematoma (Figure 3C,D). The hematoma and hygroma were evacuated by bilaterally placed burr holes, followed by an uneventful recovery.

## Discussion

With the advent of neuroimaging, there has been an increased incidence of detection of incidental asymptomatic arachnoid cysts. Arachnoid cysts most frequently occur in the middle fossa, followed by the posterior fossa, convexity, and suprasellar regions. Although these lesions are considered congenital, the exact etiology is still not clear.

Hemorrhage into an arachnoid cyst and the associated subdural hematoma following head trauma are well documented, although the mechanism and true incidence are not clearly understood. The annual risk for hemorrhage in patients with a middle cranial fossa cyst probably remains below 0.1% [2]. In a recent study by Wester et al., the incidence of chronic subdural or intracystic haematomas was reported as 4.6% of all patients with intracranial arachnoid cyst referred for treatment [3]. We propose two mechanisms leading to formation of subdural hemorrhage. First, the cyst membrane is loosely attached to the convexity dura. The mechanical forces that are sustained during a moderate head trauma can cause the cyst membrane to be detached from the dura and thus cause a bleeding episode. Second, the parietal cyst membrane also covers the area where the bridging Sylvian veins, or the veins that traverse the membrane unsupported by brain tissue, enter into the dural venous sinuses behind the sphenoid ridge. Even a moderate manipulation of the parietal membrane can disrupt these veins, leading to bleeding into subdural space [3]. Parsch and colleagues suggest an approximately 5-fold greater prevalence (2.43% versus 0.46%) of arachnoid cysts of the middle fossa in patients with chronic subdural hematomas than in the general population who undergo magnetic resonance imaging [5]. A middle cranial fossa arachnoid cyst is now recognized as one of the causes of chronic subdural hematomas after head injury, especially in young people, as the cysts appear to be more susceptible to hemorrhagic complications, including subdural and intracystic hematomas [1-8]. The membrane is vascular, and bridging veins are often observed traversing the cyst wall. This could in part explain the liability of intracystic subdural bleeding in these patients [5,6,9].

The post-traumatic hemorrhagic complication in a setting of a temporal fossa arachnoid cyst is often confined to the side ipsilateral to the cyst, and contralateral subdural hematoma is not well documented in the literature [2,8]. Occurrence of contralateral subdural hematomas with arachnoid cysts was previously reported by Mori et al. (two cases) and Parsch et al. (one case) [5,8]. Both of our patients were previously asymptomatic and had subdural hematomas contralateral to the arachnoid cyst following the head injury. This finding reinforces the notion that an arachnoid cyst, being a large fluid-filled lesion, is less compliant than normal brain parenchyma, making both ipsilateral and contralateral bridging veins prone to injury. Even though it is rare, there have been reports of ruptured arachnoid cyst, presenting with a subdural CSF collection without evidence of hemorrhage [9-11]. The sudden collapse of the cyst can cause a sudden shift of the brain, which, along with the force of the trauma, can lead to stretching, and tearing of bridging veins on the opposite side. This could explain the occurrence of contralateral subdural hematoma as in both our cases. Thus, we should inform patients with arachnoid cysts and their families of the possibility of such complications and advise care to avoid head injury in daily life, regardless of the size and symptoms of the cyst.

## Conclusion

Although many arachnoid cysts are incidentally detected and require no intervention, some of them are symptomatic. A contralateral subdural hematoma following head trauma may result from inadequate intracranial cushioning provided by the arachnoid cyst, which makes both ipsilateral and contralateral bridging veins prone to injury. It is important to identify and report such rare complications with intracranial arachnoid cysts, so that the asymptomatic patient with an intracranial arachnoid cyst can be counseled about such possibilities following head trauma.

## Abbreviations

CT: computer tomography; CSF: cerebrospinal fluid.

## Consent

Written informed consent was obtained from the patients for publication of this case report and accompanying images. A copy of the written consent is available for review by the Editor-in-Chief of this journal.

## Competing interests

The authors declare that they have no competing interests.

## Authors' contributions

PP, SKM, RPM, RK and ABP all contributed to the patients' management, the conception of the manuscript, acquisition and interpretation of data, and drafting and revision of the manuscript. DP was also involved in critically revising the manuscript and gave the final approval of the manuscript.
